# Immune Activation Signatures Associated with Fatigue in Cancer Patients Undergoing Immune Checkpoint Inhibitor Therapy

**DOI:** 10.1158/2767-9764.CRC-25-0240

**Published:** 2025-10-01

**Authors:** Howard L. Li, Soren Charmsaz, Mari Nakazawa, Stephanie L. Alden, Madelena Brancati, James M. Leatherman, Ervin Griffin, Hua-Ling Tsai, Nicole E. Gross, Christopher J. Thoburn, Alexei Hernandez, Erin M. Coyne, Sarah M. Shin, Royce P. Lee, Evan J. Lipson, Burles A. Johnson, Aliyah Pabani, Yasser Ged, Marina Baretti, Julie R. Brahmer, Jean Hoffman-Censits, Tanguy Y. Seiwert, Daniel J. Zabransky, Jennifer N. Durham, Elizabeth M. Jaffee, G. Scott Chandler, Brittany L. Adler, Won Jin Ho, Chester Kao, Mark Yarchoan

**Affiliations:** 1 Sidney Kimmel Comprehensive Cancer Center, Johns Hopkins University, a member of the imCORE network, Baltimore, Maryland.; 2 Convergence Institute, Johns Hopkins University, Baltimore, Maryland.; 3 Bloomberg∼Kimmel Institute for Cancer Immunotherapy, Johns Hopkins University, Baltimore, Maryland.; 4 F. Hoffmann-La Roche Ltd., a member of the imCORE network, Basel, Switzerland.; 5 Division of Rheumatology, Johns Hopkins University, Baltimore, Maryland.

## Abstract

Fatigue is a common and often debilitating adverse event among patients receiving immune checkpoint inhibitor (ICI) therapy, yet its underlying immunologic mechanisms remain poorly defined. We prospectively collected clinical data and blood samples from patients with solid tumors receiving ICI therapy. Patients were prospectively surveyed at month 2, 4, or 6 on treatment to assess for the presence and severity of fatigue as compared with treatment baseline. We analyzed peripheral lymphocyte populations by cytometry by time of flight and peripheral levels of 39 cytokines with a Luminex multiplex assay to identify dynamic immune changes associated with ICI-related fatigue. Of 53 patients enrolled, 31 (58.5%) reported worsening fatigue during ICI therapy. Patients reporting fatigue exhibited broad early-treatment elevations in circulating cytokines, with the most prominent increases observed in the Th1-associated cytokine cluster, including IFN-γ, IL-2, and IL-12. In parallel, several clusters of cytotoxic effector CD8^+^ T cells expanded significantly from baseline in the fatigued group. Fatigue was not associated with objective tumor response or with the development of other clinically meaningful immune-related adverse events. In a pan-tumor cohort treated with ICIs, increases in clusters of cytotoxic effector CD8^+^ T cells in parallel with related Th1-associated cytokines were associated with ICI-related fatigue, implicating fatigue as a potential marker of immune activation in this population.

**Significance:**

This study illuminates dynamic changes in peripheral cytokines and immune cell clusters that are associated with ICI-related fatigue. Namely, this study implicates the Th1 pathway as a novel contributor to ICI-related fatigue and identifies ICI-related fatigue as a clinical surrogate for immune activation in patients receiving ICI therapy. Recognizing that fatigue may be a biomarker of heightened immune activity influences monitoring strategies and informs supportive care interventions during immunotherapy.

## Introduction

Immune checkpoint inhibitors (ICI) have emerged as a cornerstone of contemporary cancer therapy. These therapies block inhibitory receptors such as PD-1 and CTLA-4, inducing a potent antitumor immune response in a subset of patients (1). However, ICIs can also cause immune-related adverse events (irAE), which can affect any organ system. Fatigue is one of the most frequent side effects reported by ICI recipients (2). Between 15% and 24% of patients receiving PD-1/PD-L1 blockade experience fatigue, and up to 40% experience fatigue after CTLA-4 blockade (3, 4). The overall incidence of fatigue in patients receiving any ICI regimen is reported to be approximately 30.4% (5). In contrast to some other irAEs, fatigue by itself is generally not life-threatening, although it can nonetheless profoundly affect the quality of life of ICI recipients through all stages of illness (6). Fatigue has been demonstrated to pose a clinically significant decline in health functioning and patient satisfaction (7). These observations underscore the need to understand the mechanisms of ICI-related fatigue and identify targets for therapeutic intervention.

Fatigue is common in patients with cancer independently of ICI treatment and is thought to result from a complex interplay between inflammation, changes in ATP and muscle metabolism, anemia, comorbidities, and cytokine dysregulation (8). Established cytokine dysregulation in cancer that may contribute to fatigue involves increased pro-inflammatory cytokines such as IL-6, IL-1, and TNF-α (9, 10). Other fatigue syndromes, such as myalgic encephalomyelitis (chronic fatigue syndrome) and cytokine release syndrome, are also associated with increased activated T-lymphocytes and pro-inflammatory cytokines such as IL-6, IL-10, and IFN-γ (11, 12). Such inflammatory cytokines have been suggested to mediate fatigue through modulation of central nervous system signaling (13). However, the mechanism through which ICIs induce fatigue in patients with cancer remains unknown.

In this exploratory study, we prospectively analyzed changes in peripheral blood cytokine levels and immune cell populations in patients with solid tumors treated with ICI therapy who reported ICI-related fatigue. We also investigated the clinical implications of ICI-related fatigue on cancer-related outcomes.

## Materials and Methods

### Study design and patient inclusion

Patients with solid tumors who received ICI treatment as standard of care at Johns Hopkins University were approached and consented for this study from May 2021 to May 2023. Clinical data were censored on April 1, 2025. Inclusion criteria included patients 18 years of age or older with pathologically confirmed solid tumors treated with ICI (including anti–PD-1/–PD-L1 and anti-CTLA-4 alone or in combination with other therapeutic agents). All patients enrolled in the study had a peripheral blood sample collected at baseline prior to initiation of the ICI. Subsequently, peripheral blood samples were collected at month 1, 2, 4, and 6 if the patient was continued on ICI and available.

Patient demographic information was obtained from the electronic medical record system, EPIC. Cancer groups for Gastrointestinal/Genitourinary (GI/GU) and other cancers are defined in Supplementary Table S1. The development of irAEs was assessed on an ongoing basis by the treating physician, and irAEs were graded based on Common Terminology Criteria for Adverse Events version 5. Investigator-assessed best responses to therapy were based on RECIST v1.1 criteria. All clinical information was reviewed by two study team members, including at least one medical oncologist reviewer.

### Fatigue survey

Patients were contacted by phone at their 2-month treatment timepoint to query for increasing fatigue since starting ICI therapy. Patients who were not successfully contacted or did not respond at month 2 could be recontacted at month 4 and month 6. Patients who were successfully contacted were asked to complete a survey to assess the impact of immunotherapy on fatigue. Patients were specifically asked, “As compared to just before you started the current immunotherapy treatment, do you feel more fatigued?” If the patients answered “yes” to the initial question, they were posed a confirmatory question to assess for a fatigue score, “If yes, how much more fatigued do you feel from a scale of 1 to 10, with 10 being the worse increase in fatigue imaginable.” Patients with fatigue scores between 1 and 5 were categorized as low fatigue, and patients with fatigue scores between 6 and 10 were categorized as high fatigue. Patients who responded that they felt more fatigued were scored as experiencing ICI-related fatigue for all analyses, whereas patients who did not note more fatigue were scored as not experiencing ICI-related fatigue. The survey was performed by an evaluator who was blinded at the time of survey to the clinical status of the patient and all correlative data.

This fatigue survey was a sub-study of the Johns Hopkins Immunotherapy biobank, a prospective effort to understand biomarkers of response, resistance, and toxicity of immunotherapy treatment. Among 103 patients prospectively enrolled in the parent study from May 2021 to May 2023, 53 patients were successfully contacted and surveyed about fatigue. No contacted patients declined to participate.

### Plasma collection

Blood samples were collected and processed as previously described by our team (14–16). In brief, venous blood samples were obtained in heparinized syringes and processed within 2 hours of blood collection. To isolate plasma, blood was transferred into a 50-mL conical tube and placed in a centrifuge at 2,000 rpm. for 20 minutes with the brakes off, and the plasma layer was isolated and stored in 1-mL aliquots at −80°C. Peripheral blood mononuclear cells (PBMC) from the remaining blood were isolated using a Leucosep tube technique. Blood diluted in equal parts of PBS was added to Leucosep tubes preloaded with Ficoll-Paque. The tubes were then centrifuged for 20 minutes at 2,000 rpm. with no brakes. The PBMC suspension was collected, and the cells were washed in PBS and stored in a cryovial initially at −80°C. This was then stored long-term in liquid nitrogen.

### Cytokine measurements

The Bio-Plex 200 platform (Bio-Rad) was used to determine the concentration (pg/mL) of target cytokines in plasma as previously described (14–16). Luminex bead–based immunoassays (Millipore) were performed following the Johns Hopkins Immune Monitoring Core SOPs and concentrations were determined using five-parameter log curve fits (using Bio-Plex Manager 6.0, RRID: SCR_014330) with vendor-provided standards and quality controls. The HCYTA-60K panel was used to detect the following cytokines: sCD40L, IL-1α, IL-1β, IL-1RA, IL-2, IL-3, IL-4, IL-5, IL-6, IL-8, IL-9, IL-10, IL-12(p40), IL-12(p70), IL-13, IL-15, IL-17A, IL-17E, IL-17F, IL-18, IL-21, IL-22, IL-23, IL-35, BAFF, G-CSF, GM-CSF, ITAC, TNF-α, IFN-γ, MCP-1, MIP-1α, MIP-1β, MIP-3α, MPIF-1, RANTES, MIG, IP-10, and VEGF-A. Concentrations which were outside of the standard curve values were categorized as “out of range” (OOR). For each cytokine, OOR below the standard curve values were replaced with the lower limit of the standard curve of the assay whereas OOR greater than the standard curve values were replaced with the upper limit of the standard curve.

### Cytometry by time-of-flight staining, acquisition, and analyses

All cytometry by time-of-flight (CyTOF) staining, acquisition, and analyses were performed at the Johns Hopkins CyTOF core facility as previously described (14–16). Briefly, patient PBMCs were thawed in a water bath at 37°C and slowly recovered using pre-warmed RPMI containing 10% FBS. Samples were counted and 2 × 10^6^ cells from each sample were plated in 96-well plates. Cells were then allowed to rest in the media for 30 minutes prior to staining. Subsequently, cells were washed once in PBS with 2 mmol/L EDTA. Cells were then incubated for 2.5 minutes at room temperature in 20 μmol/L Pt (Standard BioTools) in PBS to identify viability. Afterward, RPMI containing 10% FBS was added to the cells to quench any residual platinum. This was then washed twice with cell staining buffer (CSB; Standard BioTools). All samples were subsequently barcoded by incubating the cells with unique combinations of metal-conjugated anti-CD45 antibodies for 20 minutes. After two washes with CSB, samples were multiplexed and transferred to v-bottom flow tubes using a 40-μm filter. Each tube was then blocked using anti–human FcR block (12 μL used for 15 × 10^6^ cells) for 10 minutes at room temperature. This was followed by a chemokine stain cocktail for 10 minutes in a water bath at 37°C. Tubes were removed from the water bath, and a surface stain cocktail was added for 30 minutes at room temperature. Samples were then washed twice with CSB and fixed/permeabilized using cytofix/cytoperm solution (BD Biosciences) for 30 minutes at room temperature. These samples were then washed with perm/wash solution (BD Biosciences) and stained using the intracellular cocktail for 30 minutes at room temperature. Samples were washed twice with perm/wash solution and stored in 1.6% paraformaldehyde in PBS at 4°C until the day of acquisition, which did not exceed 1 week. On the day of acquisition, samples were stained with 1:500 ^103^Rh in Maxpar Fix/Perm solution (Standard BioTools) for 30 minutes at room temperature for cell identification. Samples were washed with PBS once and then washed and resuspended in normalization beads (Standard BioTools). The data were acquired on a Helios mass cytometer (Standard BioTools) at the Johns Hopkins University Mass Cytometry Facility. All acquired data were randomized and normalized using CyTOF software (v7.1.16389.0, Standard BioTools). Resulting fcs files were then debarcoded by manual gating using FlowJo software (v10.9.0, BD Biosciences, RRID: SCR_008520). Cell events were gated using ^103^Rh positivity. Live cells were then gated based on ^194^Pt and ^195^Pt viability staining. This was followed by debarcoding based on the positivity of unique combinations of CD45 barcodes. Each debarcoded sample was exported as an individual fcs file. Samples were normalized in R (v4.0.2) using the CytoNorm algorithm that utilized a repeated sample included in each staining batch to normalize all samples based on goal quantiles of mean marker expression. Clustering analysis was performed in R using FlowSOM (RRID: SCR_016899) to generate meta-clusters that were annotated using the expression profile of markers included in the panel. This resulted in 29 final clusters. All antibodies used for CyTOF are listed in Supplementary Table S2.

### Statistical methods

For samples collected on treatment, fold change for each timepoint was calculated relative to baseline to account for inter-patient variability. Important timepoints for our analysis included the early on-treatment timepoint, which occurred at 2 months after treatment initiation. Nonparametric methods were used to assess group differences for the cytokine analyses given skewing in the distributions; the Wilcoxon rank-sum test was used when assessing statistical differences between two groups. Log_2_ transformation to fold change was performed to reduce skewness for visualization purposes. For the CyTOF analysis, the Wilcoxon signed-rank test was used when assessing statistical differences between paired baseline and on-treatment timepoints for one group. A Fisher exact test was utilized when assessing statistical differences between two categorical variables. All statistical tests were two-sided unless stated otherwise. For the statistical analyses, *P* values < 0.05 were considered statistically significant. Statistical analysis was performed using RStudio software (version: 2024.04.0+735, RRID: SCR_000432). Only open-source software was used for this study, and no custom code was generated for data analysis of cytokine and CyTOF data.

### Study approval

This article is based upon an analysis of data collected via a standard-of-care study and using a corresponding biomarker repository conducted at Johns Hopkins University. Protocol approval was obtained by the Johns Hopkins University Institutional Review Board committee (IRB #00267960). This study was performed in accordance with recognized ethical guidelines (i.e., U.S. Revised Common Rule). All patients provided written informed consent for collection and analysis of peripheral blood samples and clinical data. In addition, patients provided written informed consent for publication during Institutional Review Board consent process. This article is sufficiently anonymized and does not contain any personal and/or medical information about an identifiable patient.

## Results

### Study cohort demographics

This study includes 53 patients who met eligibility criteria and completed the fatigue survey. The demographics of this cohort are shown in Table 1 and further described in Supplementary Table S1. Of the 53 patients, 31 (58.5%) reported ICI-related fatigue since starting ICI therapy, whereas 22 (41.5%) did not. Of the 31 patients who reported ICI-related fatigue, nine reported a high fatigue score. The cohort predominantly identified as male (66.0%), and most patients identified as White (66.0%) or Black (22.6%). The most common tumor types included hepatocellular carcinoma (22.6%), renal cell carcinoma (20.8%), and melanoma (15.1%). A majority of patients received anti–PD-1/–PD-L1 axis therapy (86.8%), with pembrolizumab as the most common ICI agent (41.5%). ICI monotherapy (39.6%) and ICI with chemotherapy and/or anti-VEGF (41.5%) were the most common treatment regimens. Most patients did not receive prior systemic therapy (67.9%) and did not have any autoimmune disease history (90.6%). No baseline patient demographic characteristics were associated with the development of ICI-related fatigue versus no ICI-related fatigue (Table 1).

**Table 1.** Patient cohort demographics with ICI-related fatigue versus no ICI-related fatigue.

Characteristic categories	Total patients *n* = 53 (%)	ICI-related fatigue patients *n* = 31 (%)	No ICI-related fatigue patients *n* = 22 (%)	*P* value
Age on study (years)	​	​	​	​
Median (range)	67 (27–83)	68 (27–80)	66 (37–83)	​
Sex	​	​	​	0.78
Male	35 (66.0)	21 (67.7)	14 (63.6)	​
Female	18 (34.0)	10 (32.3)	8 (36.4)	​
Race	​	​	​	0.31
Asian	3 (5.7)	1 (3.2)	2 (9.1)	​
Black	12 (22.6)	5 (16.1)	7 (31.8)	​
White	36 (67.9)	24 (77.4)	12 (54.5)	​
Two or more races	2 (3.8)	1 (3.2)	1 (4.5)	​
Cancer type^a^	​	​	​	0.77
GI/GU tumor	34 (64.2)	19 (61.3)	15 (68.2)	​
Non-GI/GU tumor	19 (35.8)	12 (38.7)	7 (31.8)	​
ICI treatment^a^	​	​	​	0.69
Anti–PD-1 or –PD-L1	46 (86.8)	26 (83.9)	20 (90.9)	​
Anti–CTLA-4 + anti–PD-1	7 (13.2)	5 (16.1)	2 (9.1)	​
Treatment regimen^a^	​	​	​	0.67
ICI + chemo/anti-VEGF	22 (41.5)	13 (41.9)	9 (40.9)	​
Dual ICI therapy	10 (18.9)	7 (22.6)	3 (13.6)	​
ICI monotherapy	21 (39.6)	11 (35.5)	10 (45.5)	​
Prior systemic therapy	​	​	​	0.77
Yes	17 (32.1)	9 (29.0)	8 (36.4)	​
No	36 (67.9)	22 (71.0)	14 (63.6)	​
Autoimmune disease history	​	​	​	0.68
Yes	5 (9.4)	3 (9.7)	3 (13.6)	​
No	48 (90.6)	28 (90.3)	19 (86.4)	​

Group comparisons for each characteristic were made via the Fisher exact test.

a Refer to Supplementary Table S1 for complete list of cancer types, ICI therapy type, and treatment regimen.

### Early on-treatment fatigue is associated with multiple cytokines, including Th1-associated cytokines

**Figure 1.**
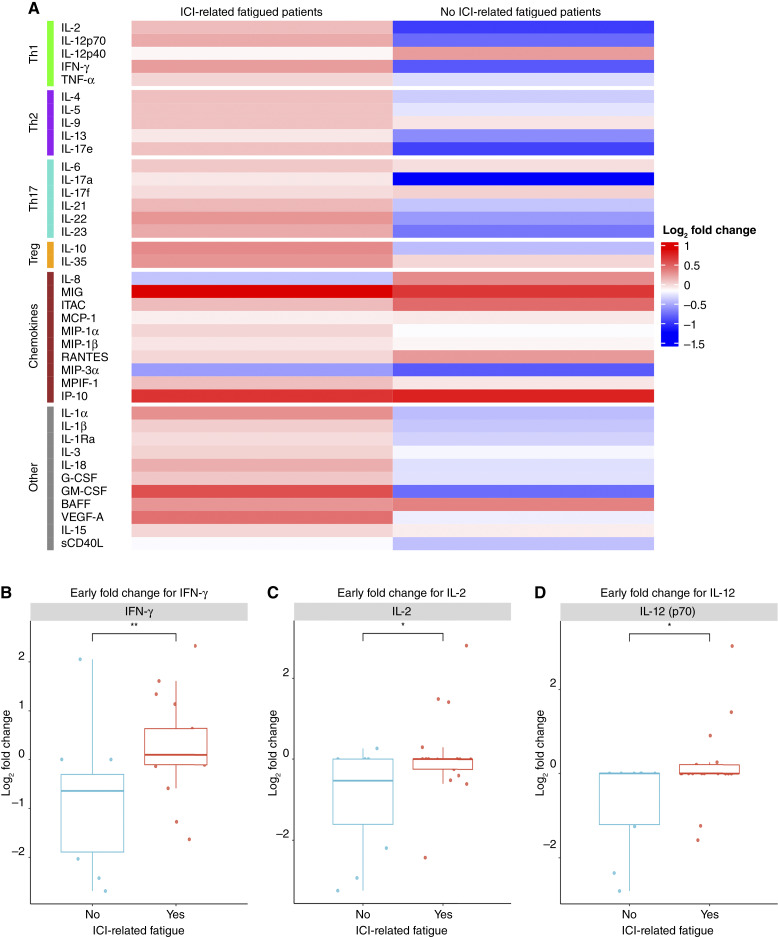
Early on-treatment log_2_ fold change differences between patients with ICI-related fatigue and no ICI-related fatigue. **A,** Heatmap of early on-treatment fold change displays a global increase in cytokines in ICI-related fatigued patients compared with non-fatigued patients. Log_2_ transformation was applied to facilitate visualization. Early on-treatment fold changes in Th1-associated cytokines were significantly increased in patients with ICI-related fatigue compared with no ICI-related fatigue, evidenced by (**B**) IFN-γ, (**C**) IL-2, and (**D**) IL-12. A Wilcoxon rank-sum test was used, *, *P* <0.05; **, *P* <0.01.

To account for the eventual heterogeneity of treatment courses, including progression, irAE development, and study attrition, the 28 patients with early on-treatment timepoints (month 2) were analyzed separately. This cohort’s demographics can be found in Supplementary Table S3. Globally, there is an overall greater increase in cytokines from baseline to early on-treatment in patients who experienced ICI-related fatigue compared with patients who did not (Fig. 1A). When comparing patients who developed ICI-related fatigue with those who did not, early on-treatment fold increases were observed across a broad range of cytokines. This was most pronounced for Th1-associated cytokines, including IFN-γ, IL-2, and IL-12 (Fig. 1B–D), which showed statistically significant elevations in the fatigued group compared with the non-fatigued group. In addition, several non-Th1 cytokines—IL-17A, IL-17E, IL-1α, and IL-22—were also significantly elevated in patients reporting fatigue (Supplementary Table S4). Beyond these specific changes, most measured cytokines increased in the fatigued group, even when individual differences did not reach statistical significance. These findings suggest that ICI-related fatigue is associated with a diffuse state of immune activation, characterized by broad polycytokine elevation, which was most pronounced for Th1-related cytokines.

We next investigated whether the severity of fatigue correlated with the magnitude of Th1-associated cytokine elevation. Although patients with higher fatigue scores exhibited numerically greater fold increases in IFN-γ, IL-2, and IL-12 compared with those with lower fatigue scores, these differences did not reach statistical significance. To assess whether the observed associations might be confounded by tumor progression, we conducted subgroup analyses stratified by disease status. Importantly, when patients with radiographic disease progression were excluded to account for cancer-related fatigue, IFN-γ and IL-12 remained significantly elevated in fatigued patients (Supplementary Fig. S1). In contrast, among patients with progressive disease, Th1-associated cytokine changes were not significantly associated with fatigue. When all patients who received ICI with chemo/anti-VEGF therapy were excluded, Th1-associated cytokine IFN-γ also remained significant (Supplementary Fig. S2). In addition, the proportion of patients with ICI-related fatigue was identical in patients who received ICI monotherapy and ICI chemo/anti-VEGF combination therapy (Supplementary Table S1). These findings suggest that the link between Th1 cytokine elevation and fatigue in ICI-treated patients is independent of tumor progression, or other agents administered in combination with ICIs, and likely reflect ICI-related immune activation.

### Cytotoxic effector T cells increase in abundance in patients with ICI-related fatigue

**Figure 2.**
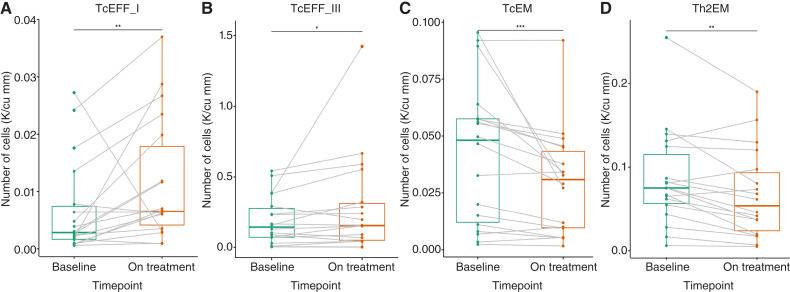
Changes in immune cell abundance in patients with ICI-related fatigue. Cytotoxic effector CD8^+^GZMB+TBET+ T cells, (**A**) TcEFF_I and (**B**) TcEFF_III, are implicated in the Th1-associated pathway and increased in abundance on treatment in patients with ICI-related fatigue. Meanwhile, effector memory T cells, (**C**) TcEM and (**D**) Th2EM, decreased in abundance on treatment in patients with ICI-related fatigue. A Wilcoxon signed-rank test was used, *, *P* <0.05; **, *P* <0.01; ***, *P* <0.001.

There were 30 patients with immune cell abundance measured at baseline and early on-treatment, of whom 18 had ICI-related fatigue. The demographics of the fatigued CyTOF cohort are presented in Supplementary Table S5. Two clusters of cytotoxic effector T cells were annotated TcEFF_I and TcEFF_III, both primarily characterized by shared CD3^+^CD8^+^GZMB+TBET+ markers. Detailed annotations of immune cell markers are detailed in Supplementary Table S6. In patients with ICI-related fatigue, TcEFF_I and TcEFF_III were significantly increased in abundance on-treatment compared with baseline (Fig. 2A and B). Conversely, effector memory T cells, annotated TcEM and Th2 effector memory, significantly decreased in abundance on-treatment compared with baseline (Fig. 2C and D). Other analyzed immune cell clusters did not have significant changes in fatigued patients. No significant differences in CD4^+^ or CD8^+^ immune cell abundances were identified in the group without ICI-related fatigue.

### Clinical response and irAEs do not definitively associate with ICI-related fatigue

**Figure 3.**
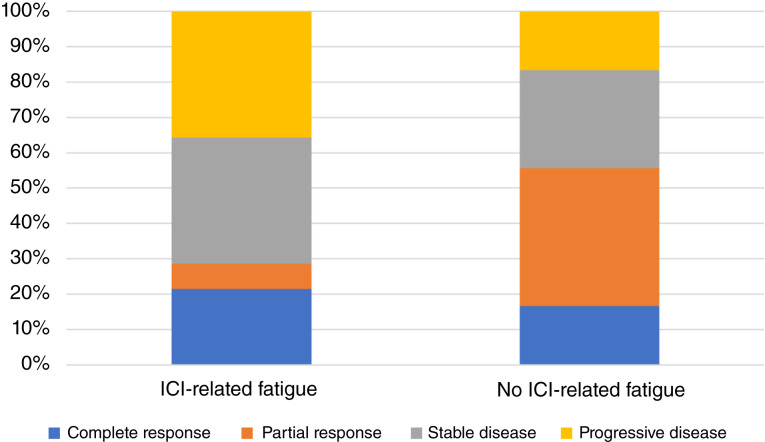
Associations between clinical outcomes and ICI-related fatigue. Stacked bar plot visualizing the best response to ICIs in patients with ICI-related fatigue vs. patients without ICI-related fatigue.

Forty-six of 53 patients had radiographically or clinically assessable best response to ICI therapy in the locally advanced or metastatic setting. The remaining seven patients had either neoadjuvant or adjuvant therapy and were not included in our clinical response analysis. The distribution of clinical responses of these patients with ICI-related fatigue versus without is illustrated (Fig. 3) and further described numerically in Table 2. Fourteen (26.4%) patients developed clinically significant irAEs (grade 2+), not including fatigue, that occurred prior to fatigue assessment or up to 1 month after (Table 3). The time period of irAE inclusion was chosen to include specifically irAEs that have the potential to affect patient-reported fatigue at the time of fatigue survey. IrAEs that occurred significantly after fatigue survey (i.e., 1+ months) were not considered to be relevant for this study. When comparing patients with ICI-related fatigue against patients without, the development of clinically significant irAEs showed no significant association (Table 3). Specific irAEs are further described in Table 4. Two patients with ICI-related fatigue and one patient without ICI-related fatigue had multiple irAEs. None of these specific irAEs were significantly associated with ICI-related fatigue compared with no ICI-related fatigue (Table 4).

**Table 2.** Best response to therapy in patients with ICI-related fatigue versus those without ICI-related fatigue.

Best response to therapy	ICI-related fatigue patients *n* = 28 (%)	No ICI-related fatigue patients *n* = 18 (%)	OR (95% CI)	*P* value
CR	6 (21.4)	3 (16.7)	1.44 (0.26–10.3)	0.72
PR	2 (7.1)	7 (38.9)	0.14 (0.01–0.87)	0.02
SD	10 (32.3)	5 (22.7)	1.43 (0.34–6.69)	0.75
Progression	10 (32.3)	3 (13.6)	2.72 (0.56–18.19)	0.2
Objective response (CR + PR)	8 (28.6)	10 (55.6)	0.33 (0.08–1.30)	0.12

The Fisher exact test was used to calculate ORs comparing patients between groups for response categories.

Abbreviations: CI, confidence interval; CR, complete response; PR, partial response; SD, stable disease.

**Table 3.** Patients with and without ICI-related fatigue who developed clinically significant irAEs.

irAE (grade 2+)^a^	Total patients *n* = 53 (%)	ICI-related fatigue patients *n* = 31 (%)	No ICI-related fatigue patients *n* = 22 (%)	*P* value
​	​	​	​	0.35
Yes	14 (26.4)	10 (32.3)	4 (18.2)	​
No	39 (73.6)	21 (67.7)	18 (81.8)	​

a Yes irAE refers to irAEs that occurred before fatigue assessment or up to 1 month after.

**Table 4.** Specific irAEs experienced by patients with and without ICI-related fatigue.

Specific irAEs (grade 2+)^a^	Total patients with irAE *n* = 14 (%)	ICI-related fatigue patients *n* = 10 (%)	No ICI-related fatigue patients *n* = 4 (%)	*P* value
Hypothyroidism	8 (57.1)	5 (50.0)	3 (75.0)	0.58
Dermatitis	3 (21.4)	2 (20.0)	1 (25.0)	1
Colitis	2 (14.3)	1 (10.0)	1 (25.0)	1
Arthritis	1 (7.1)	1 (10.0)	0	​
Pneumonitis	1 (7.1)	1 (10.0)	0	​
Hepatitis	1 (7.1)	1 (10.0)	0	​
Sicca syndrome	1 (7.1)	1 (10.0)	0	​

Percentages exceed 100% as some patients had multiple clinically significant irAEs.

a Yes irAE refers to irAEs that occurred before fatigue assessment or up to 1 month after.

## Discussion

Although fatigue is one of the most common side effects of ICI therapy, the biological mechanisms underlying ICI-related fatigue are not well understood. We examined changes in plasma cytokines and immune cell populations among ICI recipients to understand the underlying immune mechanisms of ICI-related fatigue (13). We find that ICI-induced fatigue is associated with a Th1-skewed immune response, as evidenced by increased Th1-associated cytokines, increased Th1-associated CD8^+^ cytotoxic T cells, and reduced Th2 effector memory cells. This enhanced Th1-associated response is similar to chronic fatigue syndromes in other clinical contexts, such as long COVID and myalgic encephalomyelitis/chronic fatigue syndrome, which have been found in association with enhanced IFN-γ levels (17). Collectively, these data support fatigue as a surrogate for Th1-skewed immune activation in ICI recipients.

Although we observed a relationship between fatigue and Th1-skewed immune activation—and despite prior studies demonstrating that Th1 polarization is generally associated with favorable immunotherapy outcomes (18)—we did not find a significant association between ICI-induced fatigue and clinical outcomes. Prior studies have yielded mixed results, with one study reporting improved response rates among fatigued patients, whereas another observed worse outcomes in those who experienced early on-treatment fatigue (2, 19). We hypothesize that these conflicting findings may reflect the complex interplay between fatigue, cancer status, tumor type, and other clinical variables. Notably, in our dataset, fatigue was numerically enriched both among patients who achieved a complete response and those with progressive disease, raising the possibility that fatigue may be driven by either immune activation or tumor progression, thereby obscuring its relationship with clinical outcomes.

This study has several limitations. First, cancer progression may confound the assessment of ICI-related fatigue. We attempted to mitigate this potential confounder by asking patients to specifically identify fatigue that worsened on ICI therapy in relation to a baseline of prior to ICI treatment initiation. Additionally, in a sensitivity analysis restricted to patients without progressive disease, the association between fatigue and elevated Th1-associated cytokines remained significant. In contrast, among patients with disease progression, Th1 cytokine changes were not linked to fatigue, suggesting distinct mechanisms underlying ICI-related fatigue versus cancer-related fatigue. Another potential confounder is the known association between fatigue and various irAEs, including endocrinopathies, which are independently associated with fatigue; notably, fatigue has been reported to precede irAE onset by about a week (20). However, in our cohort, worsening fatigue was not significantly associated with subsequent or concurrent irAE development. IrAE-related thyroiditis, a known contributor to fatigue, also showed no significant link to worsening fatigue in our early-treatment cohort (21). Additionally, some patients received combination therapy with ICIs and other agents, including chemotherapy, which can independently cause fatigue (5, 22). The heterogeneous pan-tumor nature of our cohort may have limited our ability to draw conclusions about treatment outcomes, given the large variability in anticipated response rates across the patients who were treated. Because of the small sample size of our heterogeneous cohort, our ability to perform sub-analyses was limited. Overall, we demonstrate that in patients receiving cancer ICI therapy, worsening fatigue associates with expansion of cytotoxic effector CD8^+^ T cells and Th1-associated cytokines, implicating fatigue as a marker of immune activation in patients treated with ICIs.

## Supplementary Material

Supplemental Table 1 Supplemental Table 1 shows cancer types and treatment regimens represented in the cohort.

Supplemental Table 2 Supplemental Table 2 shows antigens for CyTOF identification.

Supplemental Table 3 Supplemental Table 3 shows the characteristics of the early-on-treatment cohort.

Supplemental Table 4 Supplemental Table 4 shows all significant cytokines for differences in fold change between fatigue and non-fatigued patients.

Supplemental Table 5 Supplemental Table 5 shows the characteristics of the fatigued CyTOF cohort.

Supplemental Table 6 Supplemental Table 6 shows markers used to identify cell clusters for CyTOF.

Supplemental Figure 1 Supplemental Figure 1. Early-on-treatment Log2 Fold Change Differences After Excluding Progressors. After excluding patients who had disease progression within 2 months of early-on-treatment fatigue inquiry, Th1 cytokines are still significantly increased in patients with ICI-related fatigue compared to no ICI-related fatigue, evidenced by (A) IFN-gamma and (B) IL-12. Wilcoxon Rank-Sum Test, *p<0.05.

Supplemental Figure 2 Supplemental Figure 2. Early-on-treatment Log2 Fold Change Differences After Excluding Patients Receiving ICI + Chemotherapy/Anti-VEGF. Th1 cytokine, (A) IFN-gamma, remains significant after excluding patients who received ICI + Chemo/Anti-VEGF therapy. Wilcoxon Rank-Sum Test, *p<0.05.

## Data Availability

The data generated in this study are available upon request from the corresponding author.
