# Supplementary figures and images for: Immune Activation Signatures Associated with Fatigue in Cancer Patients Undergoing Immune Checkpoint Inhibitor Therapy
Source: Cancer Res Commun. 2025 Oct 1;5(10):1738–46. doi: 10.1158/2767-9764.CRC-25-0240 (PMC12485599; doi:10.1158/2767-9764.CRC-25-0240)

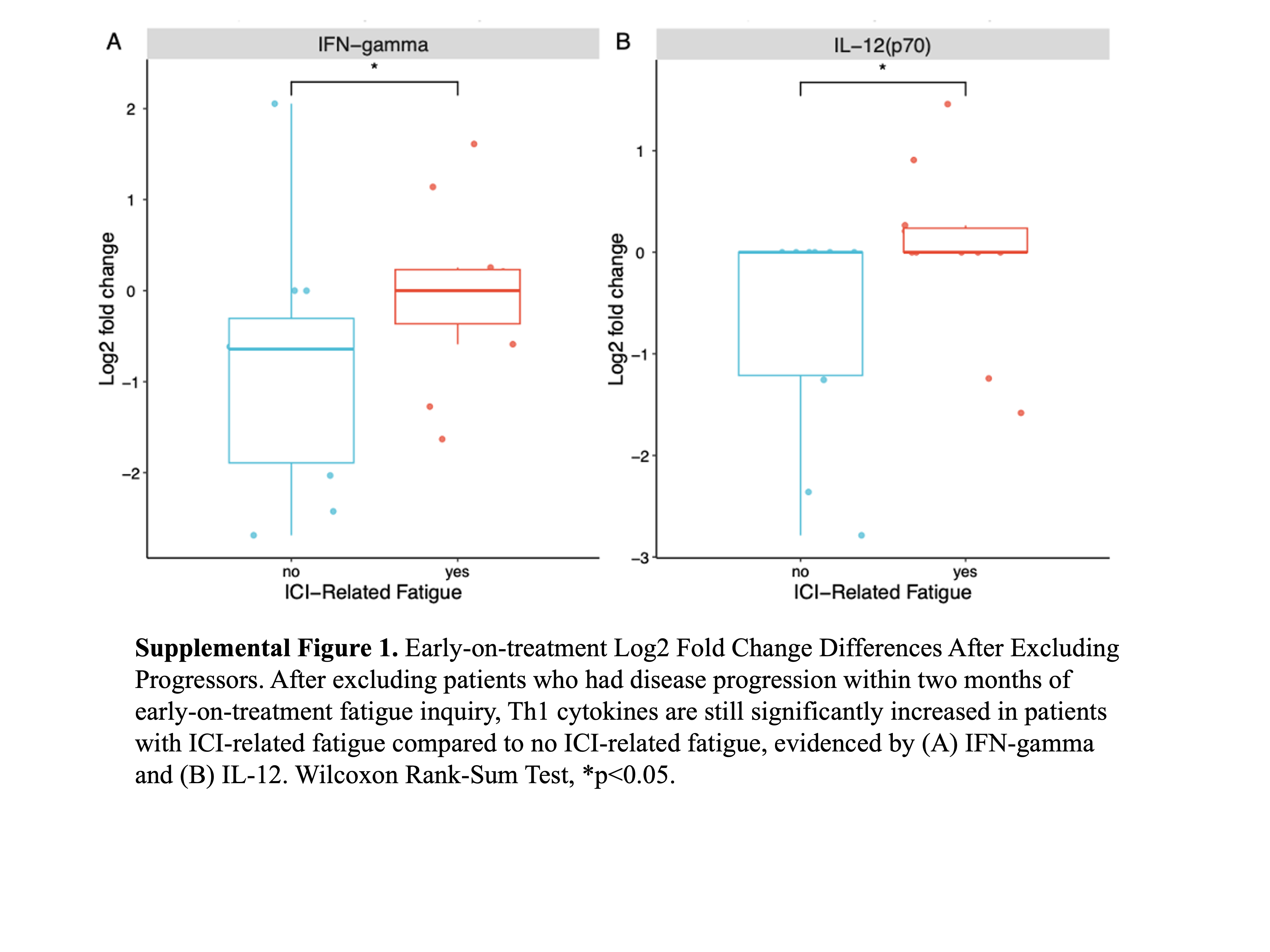

Supplement: Supplemental Figure 1 — Early-on-treatment Log2 Fold Change Differences After Excluding Progressors. After excluding patients who had disease progression within 2 months of early-on-treatment fatigue inquiry, Th1 cytokines are still significantly increased in patients with ICI-related fatigue compared to no ICI-related fatigue, evidenced by (A) IFN-gamma and (B) IL-12. Wilcoxon Rank-Sum Test, *p<0.05. [file crc-25-0240_supplemental_figure_1_suppsf1.png]

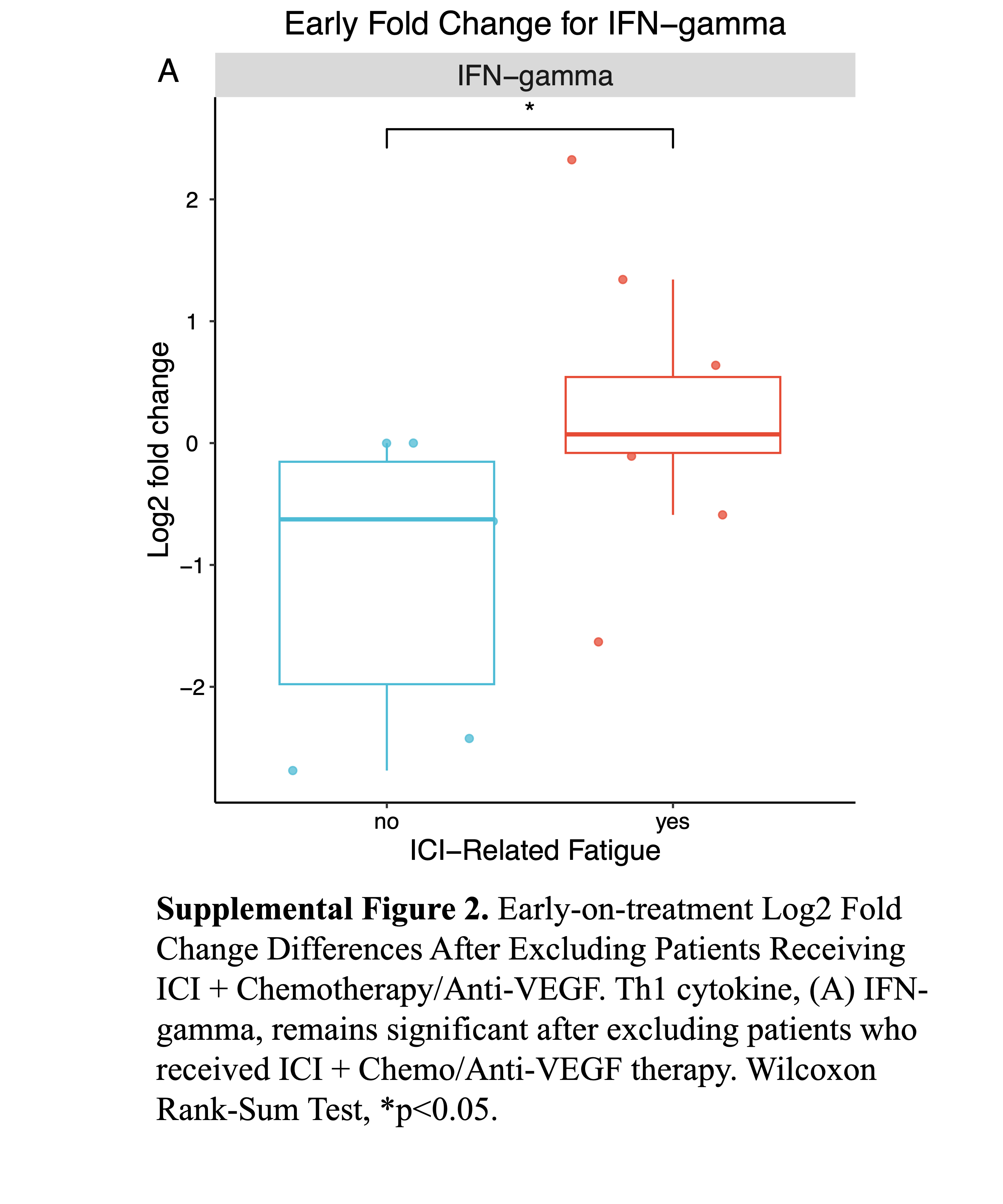

Supplement: Supplemental Figure 2 — Early-on-treatment Log2 Fold Change Differences After Excluding Patients Receiving ICI + Chemotherapy/Anti-VEGF. Th1 cytokine, (A) IFN-gamma, remains significant after excluding patients who received ICI + Chemo/Anti-VEGF therapy. Wilcoxon Rank-Sum Test, *p<0.05. [file crc-25-0240_supplemental_figure_2_suppsf2.png]
